# Eating and Drinking with Acknowledged Risks (EDAR) in Older Adults: A Qualitative Study of the Experiences of Clinicians in Japan and the UK

**DOI:** 10.1007/s00455-024-10765-4

**Published:** 2024-11-13

**Authors:** Yuki Yoshimatsu, Marianne Markowski, David G. Smithard, Dharinee Hansjee, Tadayuki Hashimoto, Hiroyuki Nagano, Ryan Essex

**Affiliations:** 1 Elderly Care, Queen Elizabeth Hospital, Lewisham and Greenwich NHS Trust, Stadium Rd, London, SE18 4QH UK; 2 Centre for Exercise Activity and Rehabilitation, School of Human Sciences, University of Greenwich, London, UK; 3 The Institute for Lifecourse Development, University of Greenwich, London, UK; 4 Centre for Chronic Illness and Ageing, Faculty of Education, Health and Human Sciences, University of Greenwich, London, UK; 5 Speech and Language Therapy, School of Health Sciences, University of Greenwich, London, UK; 6 Department of General Medicine, Osaka Medical and Pharmaceutical University Hospital, Takatsuki, Japan; 7 Department of Emergency Medicine, Brigham and Women’s Hospital, Boston, MA USA; 8 Department of Healthcare Economics and Quality Management, Graduate School of Medicine, Kyoto University, Kyoto, Japan; 9 Centre for Professional Workforce Development, Faculty of Education, Health and Human Sciences, University of Greenwich, London, UK

**Keywords:** Dysphagia, Swallowing impairment, Aspiration pneumonia, Decision-making, Risk feeding

## Abstract

Eating and drinking are fundamental to life. However, older patients are often restricted with oral intake due to feared risk of aspiration. Eating and Drinking with Acknowledged Risks (EDAR) is an alternative process which enables comfort, dignity, and autonomy for these patients. While national guidance has been developed for EDAR in the UK, other ageing societies such as Japan do not have such guidance. To understand the perspectives and experiences of healthcare professionals regarding the complex decision-making process around EDAR we planned a mixed methods study comparing the two countries. This was the qualitative phase of the study. Twelve healthcare professionals (two doctors, nurses and speech and language therapists each in Japan and the UK) participated in semi-structured interviews on their roles and experiences related to EDAR in older adults. We analysed the data thematically, and three themes emerged: (1) healthcare professionals and healthcare systems, (2) priorities in decision-making and (3) relationship with family and patient. There were many similarities but also differences across the countries, in the setting, training and individual experience. Decision-making was shaped by a complex combination of individual, structural and cultural factors, which indicated in the Japanese culture a greater likeliness to defer clinical decision-making and to side with families’ wishes. Healthcare professionals’ experiences and attitudes towards EDAR differed depending on various factors related with the individual and environment. The next quantitative phase of our research aims to establish the mechanism to increase confidence around EDAR in professionals and training options.

**Supplementary Information:**

The online version contains supplementary material available at 10.1007/s00455-024-10765-4.

## Introduction

Eating and drinking are basic human rights and a fundamental joy in life. When there is a suspected risk of aspiration or choking, clinicians generally tend to restrict people from eating and drinking in the hope to prevent aspiration [1, 2]. This happens particularly often in older adults, associated with a high prevalence of dysphagia and aspiration pneumonia.

However, restricting eating or drinking does not necessarily prevent aspiration pneumonia (which is more of a multifactorial syndrome than a simple result of dysphagia alone [3]), while it will potentially contribute to malnutrition, dehydration, oral frailty, and deprivation of basic human rights and a decline in quality of life [2, 4].

Eating and Drinking with Acknowledged Risks (EDAR) is an alternative route that enables comfort, dignity, and autonomy for patients who may otherwise be restricted with oral intake [5]. In the UK, the Royal College of Speech and Language Therapists have published guidance [5]. The Royal College of Physicians and the British Geriatric Society have also published practical guides on the complexities of nutrition and hydration in older adults and end-of-life care [6]. These guidances have shown to help promote EDAR [7, 8], and the Feeding via the Oral Route With Acknowledged Risk of Deterioration (FORWARD) bundle has been developed and tried in stroke patients, to enable early start of oral intake [7]. However, decision-making around EDAR implies that the clinician has to consider medical and ethical factors, and this can lead to potential conflicts and risk-taking. As the risks and benefits of eating and drinking cannot easily be quantified, the decision-making process is complex, with added ethical dilemmas to consider [9]. How to make the process easier is largely unknown, which is why we planned a mixed methods study to better understand the current healthcare professionals’ perspectives in order to consider what interventions would be of use. A qualitative study in New Zealand identified four global themes surrounding EDAR: supporting practice, communication, complexity of feeding decisions, and patient and family centred care [10]. Concerns were raised towards limited education and organisational policy around decisions, and communication was considered a major factor in the success [10]. In the UK, there is a general emphasis towards an individual’s comfort. This may be due to cultural and religious factors and the wide acceptance towards geriatric and palliative care. With the recent development of guidelines, it has become more common to consider EDAR in practice. Despite these advantages, EDAR is not an easy decision for clinicians in the UK [1, 11].

In countries such as Japan, there is still no such policy regarding EDAR. Japan has ranked top in longevity for most of the past decade [12]; however, their healthy life expectancy is 10 years less than biological life expectancy [13]. Although Japan leads the world in the abundance of modified diets [14] and oral care availability [15], there is a tendency to prioritise safety over comfort, and as a result, many people are put on nil by mouth (NBM) when suspected to be at high risk of aspiration [2, 16]. Many older adults spend their final months/years with high dependency needs and little or no oral intake, being given nutrition via intravenous drips [17], peripherally inserted central catheters [18] or tube feeding [19]. These measures are said to prolong life expectancy in Japanese studies [19, 20], though data from Western countries state otherwise [21, 22]. Moreover, longevity and families’ wishes are often emphasised over patient comfort or autonomy. The Japan Geriatrics Society issued a guideline on end-of-life care for older adults, suggesting the shared decision-making of withholding or withdrawing artificial hydration and nutrition [23]. However, there is no formal assessment of capacity that is routinely performed in the clinical setting, nor practical guidance on how to approach decision-making on EDAR.

To assure older adults of their rights to enjoy eating and drinking while minimising the burden on clinicians, it is necessary to understand the medical, cultural, and ethical issues related to EDAR in each setting. Therefore, based on these cultural and medical differences in the two super-ageing societies Japan and the UK, we planned a mixed methods study (exploratory sequential design) [24] on EDAR in older adults comparing the two countries. The primary aim was to investigate the barriers and facilitators healthcare professionals face when considering EDAR for older adults, with the secondary aim being to compare the similarities and differences in UK and Japan, and deliberate what can be done to promote EDAR. As no similar study has been performed, we conducted an initial feasibility study [11] and qualitative phase, which would then lead to the quantitative phase of developing a questionnaire for healthcare professionals to take part.

Here, we report the qualitative phase of the study to explore the views of clinicians in Japan and the UK toward EDAR and the factors that shaped their decision-making in relation to this practice.

## Methods

This was a qualitative study on healthcare professionals regarding decision-making around EDAR in older adults. Ethical approval was provided by the Ethics Committee of Hashimoto Municipal Hospital in Japan (ID R2.10-1) and the Health Research Authority and Health and Care Research Wales in the UK (ID 321158). Written informed consent was obtained from each participant. The objectives and nature of the study were fully explained with written and oral explanation. Potential participants were given the opportunity to ask questions. Participants were able to withdraw consent at any time.

### Participants and Recruitment

Twelve multidisciplinary healthcare professionals working clinically with patients aged 65 years-old and above in Japan and UK were recruited. There were 2 representatives from each professional group comprising doctors, nurses, and speech and language therapists (SLTs), who form the three key professions involved in the decision-making around eating and drinking in both countries. The number of interviewees were kept to a minimum as this qualitative study was an exploratory phase leading to the quantitative phase: therefore; the emphasis was placed on the identification of themes from a spread of participants purposively recruited, rather than exploring a topic in depth by a larger number of participants (the latter will be achieved with the second phase). The contested concept of data saturation is not relevant to our research design as we aimed to achieve ‘conceptual depth’ [25], where we focused in particular on the ‘range’ (breadth of issues) between the two countries.

Subjects were recruited through purposive sampling. The inclusion criteria were healthcare professionals who have worked in Japan or the UK for 5 or more years and are currently involved in the management of older adults with dysphagia. There was no limitation to their age, gender, or ethnicity.

The demographic data of the interviewees are shown in Table 1. The UK interviewees were of varied ethnicity.

**Table 1:** Demographic data of interviewees

ID	Country	Occupation	Setting	Experience (years)	Age (years old)	Sex
JPN1 S	Japan	SLT	Acute hospital / community	24	50	F
JPN2 P	Japan	Physician	Acute hospital / community, general medicine	13	36	M
JPN3 N	Japan	Nurse	Acute hospital / community	15	43	F
JPN4 S	Japan	SLT	Acute hospital / community	11	33	M
JPN5 P	Japan	Physician	Acute hospital, respiratory medicine	28	60	F
JPN6 N	Japan	Nurse	Acute hospital	20	42	M
UK7 S	UK	SLT	Acute hospital / rehabilitation ward / community	14	36	F
UK8 S	UK	SLT	Acute hospital / community	11	39	F
UK9 N	UK	Nurse	Acute geriatric ward	20	52	F
UK10 N	UK	Nurse	Acute geriatric ward	11	37	F
UK11 P	UK	Physician	Acute hospital, geriatric medicine	15	43	M
UK12 P	UK	Physician	Acute hospital, geriatric medicine	34	58	F

(F: female, M: male, SLT: speech and language therapist)

### Interviews

A semi-structured interview topic guide was developed by researchers (Supplementary 1). Questions were designed to answer the research objectives and find the barriers and facilitators of decision-making around EDAR. A multidisciplinary team of researchers revised the questions. All interviews were conducted by YY in a quiet room. The duration ranged from 20 to 50 min and were recorded, then transcribed within 1 week after the interview. The Japanese interview data was translated into English by YY and was individually checked for accuracy by two other bilingual researchers (TH and HN).

### Analysis

Data was analysed thematically in NVivo (version 14) [26], following the steps laid out by Braun and Clarke [27]. Each transcript was reviewed by at least two authors in arriving at our final themes. The following steps were taken: (1) Familiarisation with data. RE, MM and YY familiarised themselves with the data by reading through transcripts and taking notes. (2) Coding the data. RE generated a series of initial codes, going through each transcript line by line, inductively identifying concepts and ideas that emerged. (3) Generating themes from the codes. RE generated initial overarching themes from the codes. (4) Reviewing themes for coherence and relevance. At least two authors reviewed each transcript independently, and three authors critically analysed coding. RE, MM, YY critically reviewed the initial codes and identified emerging themes. (5) Defining and naming themes. Through an iterative process, where disagreements and divergence were resolved through discussion, RE, MM, and YY arrived at the final themes (Table 2). To ensure rigor, the final themes were reviewed independently by four colleagues (two each from Japan and the UK) experienced in decision-making in eating and drinking in older adults. (6) Writing up the analysis and reporting the findings.

## Results

Three overarching themes emerged from the data: healthcare professionals and healthcare systems, priorities in decision-making and relationship with family and patient (Table 2). Each of these themes, along with their main sub-themes will be discussed below.

**Table 2:** Themes and subthemes extracted from the interview data

Themes	Subthemes
Healthcare professionals and systems	Decision-making approaches
	Experience with EDAR and training
	Hospital or community settings
Priorities in decision-making	Clinical indicators
	Patients and families’ wishes
	Risk, regulatory concerns, and ethical dilemmas
Relationship with family and patient	Educating and supporting patients and families
	Trust and time

### Theme 1: Healthcare Professionals and Healthcare Systems

Beliefs of healthcare professionals were important factors that impacted their views toward EDAR, as were the healthcare systems in which they worked. Three sub-themes emerged related to their views about who should make the decision about EDAR: their experience with EDAR, the use of checklists and training opportunities. Participants also spoke about the relationship between the systems setting (community or hospital) and what effect this had in terms of applying EDAR.

### Decision-Making Approaches

There was substantial variation in the answers to the question of who had the final say in EDAR. Several participants spoke about deference of decision to senior staff, often the SLT or consultant who oversaw the care of the patient. Generally, there was more acceptance amongst Japanese clinicians to defer decision-making to senior staff.

However, some Japanese participants expressed wanting to have more say in regard to the patients care.

Currently, it feels like a top-down approach from doctors. Nurses are also not able to speak up because their responsibilities are not clearly defined, leading to ambiguity. (JPN5 P)

In contrast, it appeared that clinicians from the UK were more likely to take decisions by themselves and then involve others in confirming their decision.

I mainly make the decision by myself, but would involve the person, the family, the carers. A lot of times I would also let the GP know and ask them to contact me if they don’t agree. (UK8 S)

Several participants, more so from Japan, called for a more collaborative approach. This varied from simply wanting to have a “second opinion” (UK10 N) to having more broad ranging interdisciplinary discussions.

Instead of just doctors and nurses getting together to talk, it would be better to have a team where experts can discuss cases. No matter how many different professions talk together, if they don’t have the right knowledge and experience, the basis for the decision-making won’t be clear. (JPN2 P)

For some in both countries, this support was already in place, with a few participants speaking about having access to specialist teams and nutrition nurses.

Furthermore, clinicians from Japan often spoke about having no clear procedure or protocol for EDAR. One nurse participant noted that “[t]here is no unified method. We are always struggling and exploring” (JPN3 N). This lack of clarity often created confusion.

Depending on who is in charge, sometimes the patient can eat and sometimes they can’t. It’s a big problem because although swallowing function does affect the decision, the decision is largely influenced by how the clinician thinks. Whether someone can eat or not depends on who is managing their care. (JPN1 S)

Several participants noted that they did not have any guidelines in place, with only one participant reflecting on the fact that a protocol was utilised in their workplace. Views about guidelines were mixed. A number of participants in both countries felt that uniform guidance could help improve practice and fill professionals with more confidence concerning potential legal repercussions.

Others were more measured in seeing guidelines as a baseline measure that could provide minimal standards, rather than a solution that would improve practice.

Guidelines and policies are absolutely necessary to know the process to follow and how to make the decision. But so much of it is not covered in them. When the patients say I’m not doing that, and they’ve made that decision, then what do you do? (UK8 S)

A number of participants were more overtly sceptical about the utility of guidelines when it came to EDAR since professionals must also deal with the patient’s and families’ emotions. At least one participant spoke about the difficulty of following guidelines in this context, while others felt that guidelines provided little in the way of empathy.

I think it is difficult to show compassion while using a set of uniform phrases provided as guidelines. It is important to reconcile the medical staff with the patients and their families to see if there are any gaps in their understanding of their condition. (JPN2 P)

### Experience with EDAR and Training

Importantly the extent to which participants felt they needed the support of a team or other specialists was often shaped by their experience with and confidence related to EDAR.

A number of participants spoke about lack of education and their initial inexperience with EDAR and how, in lieu of more formal training, they learnt on the job. This was common amongst clinicians from both countries.

I learned by talking to nurses, patients and families, and dealing with them individually. There were no fellow SLTs around, so I wasn’t able to share experiences with people of the same profession. (JPN1 S)

The more you experience, the more proficient you are, and the decision comes almost automatically. That’s how you learn. Practice. (UK10 N)

While a number of participants reflected on the experience that accumulated with years of practice, many more spoke about specific influential experiences which had prompted them to shift their own practice.

My training at a well-established hospice also had a significant impact on me. There, I learned a great deal about end-of-life care. Nurses would sit by the patient’s side, holding their hand without any tubes stuck to their bodies. Observing this approach to supporting patients made me realise that this kind of care is wonderful. (JPN5 P)

Participants from both countries reflected that this lack of preparedness may be because of the broader focus of medicine to be curative, rather than on death and dying.

In medical school, we learn how to save lives, but we don’t learn about dying. It’s understandable since medical care is primarily focused on preserving life. However, taking care of patients towards the end of their lives is also part of a doctor’s job, so I believe it’s important to teach these aspects as well. (JPN5 P)

While at least one participant noted they had training on EDAR “regularly” (UK10 N), the majority of participants spoke about the need for more training, particularly in end-of-life care.

Participants pointed to the importance of experience to complement education and implied that for many difficult decisions, education alone may not be the best preparation, but needed experience with practice.

### Hospital and Community Settings

A number of participants spoke about the potential burdens in relation to EDAR, mainly on staff and already strained healthcare systems. Similar sentiments were shared by UK and Japanese participants.

There is a concern that when we try EDAR in the hospital, then there will be more pneumonia recurrences, prolonging hospital stays. (JPN2 P)

Staff need to have the time, recognition, and support to be able to spend time with patients and make appropriate assessments and decisions. (UK9 N)

While most concerns related to patients who were hospitalised, participants were also concerned about patients in community settings. Participants noted the need for greater time and resources as they related to upskilling staff in relation to EDAR in the community.

The question of the setting was often critical in shaping participants’ perceived risks of EDAR and how they made decisions. Most participants felt that care was more risk averse in hospitals. One felt that making decisions about EDAR was easier in hospital, because of the support and “immediate medical care” (JPN4 S) that was available.

The responsibility that came with EDAR in the community appeared to be particularly felt by Japanese clinicians.

In the hospital, it was left to other professions, but at home, we have to do it ourselves, so I feel a strong sense of responsibility and caution; I need to be very cautious. (JPN3 N)

Despite this however, many still felt the community was the best place to promote EDAR as clinicians can work more closely with patients’ wishes and with family support towards improved quality of life.

### Theme 2: Priorities in Decision-Making

The second theme relates to what participants prioritised in their decision-making in relation to EDAR. There were three sub themes in this theme: clinical indicators (such as the patients’ ability to swallow), the patients’ and families wishes, the clinicians’ perceptions of risk, legal and regulatory concerns and their values around EDAR.

### Clinical Indicators

Clinical assessment was often the first step taken towards EDAR. This could range from informal conversations about what patients could eat, to formal assessments involving multiple team members.

Critical in this assessment was forming some idea of the patients’ risk related to choking.

If we know that somebody is a big choking risk, if patients still want to eat, it’s important to make sure they have a crisis plan in that event. (UK7 S)

Overall, participants spoke about such assessments as one factor in a relatively complex puzzle that involved the input of families, perceptions of risk and their own ethics or values.

### Patients’ and Families’ Wishes

The role and influence patients and families have on decisions related to EDAR was discussed widely. Both participants from Japan and the UK spoke at length about prioritising patients’ wishes and why these were so important.

It is necessary to listen carefully to the patient’s various thoughts and feelings, not just their desire to eat. (JPN3 N)

What does the patient want, that is my priority. And if the patient wants to eat, rarely would the family disagree. (UK9 N)

For a number of participants, respecting patient and family wishes were closely entangled with respect to the patient’s autonomy and their quality of life.

Patients and their families are instantly brighter when they are allowed EDAR. Eating and drinking also makes them more positive about other things. I feel that instead of patients staying in bed all day and thinking there’s nothing more to live for, when they have the choice to eat or drink, they spend more time awake and are more positive about other things that go along with eating. (JPN3 N)

In practice however, this was not always straightforward, particularly when there were conflicts between patients and their families. Many spoke about how mediating such disputes was not simple. Particularly, Japanese clinicians were inclined to side with the family despite their intentions to fulfill the patients’ wishes.

I really want to respect the patient’s wishes, but a lot of the time I find myself siding a bit with the family’s wishes. This is because it’s the family who are closest to the patient. If the family cannot be positive and do things properly, no matter how much the patient wants it, it won’t work. (JPN3 N)

### Risk, Regulatory Concerns and Ethical Dilemmas

Participants spoke about the risks related to EDAR and each weighed the risks differently. While the views largely varied, there was a tendency for UK participants to be more confident about their decisions and be more willing to take risks, while Japanese participants appeared to have a more risk-averse behaviour.

One participant noted that if there were any risk, they generally would not feed a patient, while another felt that it was far more acceptable now to take such risks, at least in the UK.

Risk of aspiration was specifically cited a number of times.

The primary concern is the risk of aspiration. Aspiration can be life-threatening, and even if it doesn’t result in death, it can lead to pneumonia and cause significant distress to the individual. Considering this, it is essential to proceed with caution. (JPN4 S)

It would be awful for someone to choke to death. And no matter their understanding, I think that would be an awful way to die. (UK7 S)

One further factor that influenced risk-taking was related to the ability to modify the food.

I try to modify the food down to a safe level as much as possible, so that they can enjoy it. I don’t push them away as an instant no, but I can’t grant their wishes per se. (JPN1 S)

One further risk that deserves special attention is related to legal or regulatory sanctions. How they influenced participants’ decision-making differed substantially. Participants spoke about the need and importance to have mutual understanding with families in regard to EDAR.

I have never had to worry about being sued or anything like that. There have been conflicts, and it took time to understand each other, but in the end, there is often mutual understanding. (JPN5 P)

At least one participant had been through legal proceedings already. This had an ongoing influence on their approach to EDAR.

I’m not really sure what my legal standpoint is. There is a chap that is a high risk. I’ve done letters to everybody, etc. I’ve done everything I can think of. But am I going to end up in court because his condition changes? or if someone does something that doesn’t work on that day? Is that my fault? should I go back and assess him again? I ask myself so many questions. (UK8 S)

Participants further spoke about EDAR in regard to their own or their professional ethics. The idea of ‘doing no harm’ was mentioned by at least two participants, who felt by allowing EDAR they may have been promoting harm.

If I know that the swallowing function is that bad and that my decision [allowing EDAR] is going to harm the patient, it’s a dangerous thing to do. That would mean that my swallowing assessment is not done efficiently. I would say to myself, how is that professional? (JPN1 S)

Importantly, participants did not only speak about ethics in relation to limiting EDAR; many spoke about the importance of valuing patient autonomy and how this directed their decisions, while other spoke about the de-medicalisation of death.

I think we need to stop medicalising death or end-of-life and concentrate on patients’ wishes and what they would want. (UK12 P)

### Theme 3: Relationship with Family and Patient

The relationship with family and patients was also critical in shaping decisions in relation to EDAR. Two sub-themes emerged here, educating and supporting families by involving them in feeding or care, and the need for trust and time.

### Educating and Supporting Patients and Families

Patient and family’s knowledge and willingness to learn about EDAR were important factors in decision-making in providing EDAR.

I have to make sure that the family understands that the patient is not really in a position to eat, but that everyone is working together, to best fulfil the patient’s wishes. (JPN3 N)

It’s important to educate the family beforehand about the natural progression of the disease, and what to expect. This education is crucial for the family rather than the patient themselves. We need to differentiate between patients with full consciousness and those with conditions like dementia or frailty. In either case, patients should be the centre of the decision. (JPN5 P)

One common issue raised in both countries was the need for greater support for families to accept the illness of their loved ones and who might already be grieving. One participant described what they usually say:

It’s not you that’s harming them. You’re doing the best you can. It’s their dementia that’s changing the way they’re doing things. It’s not you that caused this. (UK8 S)

One further important factor was the extent to which families could engage in their care. This appeared to be more salient for Japanese participants as care is frequently delivered at home, yet families need to be educated about EDAR.

One effective method as part of family education is to ask the family to feed the patient. (JPN5 P)

It’s not easy for carers to take time to prepare and feed them in the way that the specialists advise them to, so it depends on the family. The burden on the family can be quite significant. (JPN3 N)

### Trust and Time

Finally, trust and time were critical factors that shaped the ability of participants to develop relationships with patients, their families and ultimately how they approached EDAR.

Ultimately, I think it’s about how to build trust with the patient and their family. With that, a decision can be made on EDAR. I don’t think that’s really something you make in writing [by consent forms]. In the past, we didn’t have the luxury of spending time building trust with patients and their families, but nowadays there is a much smaller number of patients per day, so I think we have more time. It’s important to have more time to build trust, to consider various modifications to food textures, to talk with the family, etc. You also need time to talk with the patient. I want more time to face the patient, not only for the legal stuff or documents. (JPN1 S)

While Japanese participants spoke about having the time and ability to build trust with patients and families, this was not always the case, particularly for UK participants.

If we had more time to spend with the patients or less caseload that would be so beneficial. Especially in the elderly care ward where the patient needs more attention, time and care. More workforce would be important. (UK10 N)

[EDAR discussions are] really time consuming. Because they aren’t 2-minute discussions. And sometimes it’s not just one discussion but a few, until you reach the point that, yes, we are in agreement. Because you approach the subject, they need to think about it, they’ve never heard of it, they need to know about the options, and they will take it back home, they will speak to the entire family, they will come back, but it takes 2–3 days or even longer, and more family come, and we explain again… (UK12 P).

## Discussion

The results summarise the challenges clinicians in Japan and the UK face in applying EDAR. A number of factors shaped attitudes and subsequent decision-making. Views about who should make decisions, the extent to which this should be collaborative and the need for guidelines varied; these were often shaped by training and experience in EDAR. Notably many participants reported limited training related to EDAR, with most learning on the job. The systems in which people worked also shaped their views, with notable differences between those in the community and those in hospitals. Perhaps unsurprisingly, many described existing pressure on staff, making EDAR more difficult. How clinical and other factors were prioritised also impacted decision-making. This included the clinicians’ perception and priority given to clinical indicators, families’ wishes and legal risk. Importantly, even where clinicians expressed a desire to honour patient autonomy, other factors may have weighed more heavily in their decision whether or not to undertake EDAR, like potential legal risk. One final factor that impacted decision-making related to the clinician’s relationship with family, with many discussing the importance of involving families in the process and the need to develop trust.

This study revealed many similarities among Japan and the UK, along with differences across the countries but also setting (hospital or community), occupation, and individual experience, as a whole painting a complex picture that speaks to how individual, structural and cultural factors shaped decision-making in this context. The main difference overall between the countries appears to be that Japanese clinicians are more likely to defer clinical decision-making to senior staff and/or to side with families’ wishes. This is in contrast to the UK, where shared decision-making approaches appear to take place more frequently, with an individual clinician leading the decision but getting confirmations from colleagues. Despite the UK having national guidance, there were similar challenges when considering EDAR. Guidance alone is not as advantageous: combination with practical knowledge and experience is what is necessary.

One interesting finding is how EDAR was frequently associated with end-of-life care in both countries, while the initial EDAR guidance aims for autonomy in eating and drinking regardless of the disease stage [5]. This was also seen in a retrospective study in the UK [11]. While there may be a need to increase awareness of the importance of autonomy in earlier disease stages, this also shows how in reality, EDAR and comfort measures are most sought for in end-of-life care. Hence, this indicates a generic need for more training and confidence with end-of-life care and support for patients and families at this crucial stage, considering the poor prognoses of older adults with aspiration pneumonia/dysphagia [11, 28–30].

The themes from our data were similar to a study of an inpatient setting in New Zealand [10], suggesting similar difficulties and dilemmas are experienced internationally. However, our study compared Japan and the UK, two culturally and religiously differing but similarly developed and ageing societies. This also allowed comparison of having a national guidance (UK) or not (Japan). Our study involved participants from both the hospital and community setting, revealing different struggles according to the setting. Some participants were more risk averse in the inpatient setting due to hospitals prioritising cure over comfort, while others mentioned inpatient settings to be beneficial for multidisciplinary involvement and medical action to be taken in emergencies. These differences are important considerations towards support and training tailored to each culture and setting; for example, hospital staff may benefit from reassurance on the justification of end-of-life comfort care while community staff may find more specialist input to be helpful.

### Implications

This study has many implications. In clinical practice, these findings highlight the importance of holding multidisciplinary discussions in these difficult decision-making processes, which would benefit patients and families but also healthcare professionals themselves by means of enabling more confidence. For policy makers and academic organisations, developing guidelines are also of importance. Nevertheless, national guidelines do not solve all issues; rather, local protocols or checklists and handouts may also be useful in the actual application of EDAR. Additionally, case-based support by specialists and multidisciplinary teams are helpful approaches. The importance of including end-of-life-care in education and training for early-career professionals is also suggested. As for research implications, being an initial exploratory study, this warrants the need for a quantitative study to further understand the barriers and facilitators of EDAR in a wider audience.

### Strengths and Limitations

This study has limitations owing to the study design of comparing just two countries, and a selection bias due to the sampling approach. The themes may not be generalisable; however, the design and sample size were selected as an initial exploratory phase to shape the following quantitative phase of the project and participants were invited from various backgrounds and experiences to maximise the variety of results. Qualitative intercultural comparison regarding clinicians’ experiences with EDAR is, to our knowledge, the first to be reported. We will conduct a quantitative phase to further investigate the barriers and facilitators of enabling EDAR in older adults.

## Conclusions

Healthcare professionals’ experiences and attitudes towards EDAR differed depending on the country, setting, profession and individual values. To further enable EDAR for appropriate candidates and increase confidence in healthcare professionals, support tailored to the setting and profession will be vital. This highlights the value of this study and warrants further quantitative investigation.

## Electronic supplementary material

Below is the link to the electronic supplementary material.

Supplementary Material 1

## Data Availability

All data are applicable in the paper.
