# Supplementary material for: Eating and Drinking with Acknowledged Risks (EDAR) in Older Adults: A Qualitative Study of the Experiences of Clinicians in Japan and the UK
Source: Dysphagia. 2024 Nov 13;40(3):650–9. doi: 10.1007/s00455-024-10765-4 (PMC12145317; doi:10.1007/s00455-024-10765-4)
Supplement: Supplementary file 1 — Supplementary Material 1 [file 455_2024_10765_MOESM1_ESM.docx]

**Supplementary 1. Interview topic guide**

1. As a healthcare professional, how do you feel about eating and drinking at the end-of-life for your patients?

2. What specific training have you had about Eating and Drinking with Acknowledged Risks (EDAR) and its implementation? How long ago was that? Is there anything that you found especially helpful? Is there anything you would have liked to learn about?

3. What process do you follow when considering EDAR?

4. What helps you to make decisions about EDAR? How do you feel about making these decisions?

5. What do you think are key factors to consider when a person wants to EDAR?

6. When deciding on EDAR, what do you think would help you to make better/more informed decisions? What support do you need? What has been un/helpful?

7. What are your concerns when a patient wants to EDAR? Who do you go to with these concerns?

8. Do you think EDAR is beneficial? why? Why not?

9. Do you discuss EDAR with other professionals/non-healthcare workers? If so, who? Why? What?

10. Is there anything you would like to tell us about EDAR that is important to know about?
